# Hydrocarbon-Based Statistical Copolymers Outperform Block Copolymers for Stabilization of Ethanol–Water Foams

**DOI:** 10.1021/acsami.2c09910

**Published:** 2022-08-19

**Authors:** James Jennings, Rebekah R. Webster-Aikman, Niall Ward-O’Brien, Andi Xie, Deborah L. Beattie, Oliver J. Deane, Steven P. Armes, Anthony J. Ryan

**Affiliations:** Dainton Building, Department of Chemistry, University of Sheffield, Brook Hill, Sheffield S3 7HF, South Yorkshire, U. K.

**Keywords:** statistical copolymers, polymeric surfactants, surface activity, foam stabilization, micellization, X-ray scattering

## Abstract

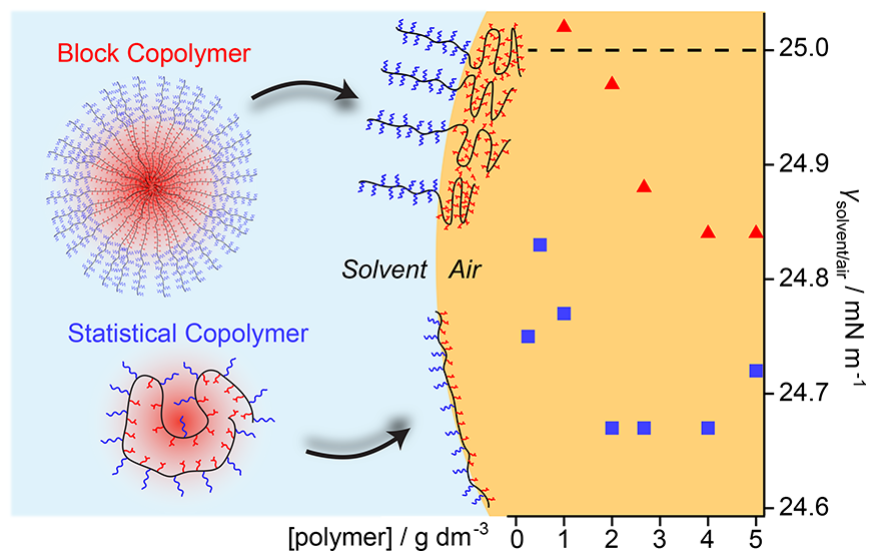

Well-defined block copolymers have been widely used as emulsifiers, stabilizers, and dispersants in the chemical industry for at least 50 years. In contrast, nature employs amphiphilic proteins as polymeric surfactants whereby the spatial distribution of hydrophilic and hydrophobic amino acids within the polypeptide chains is optimized for surface activity. Herein, we report that polydisperse statistical copolymers prepared by conventional free-radical copolymerization can provide superior foaming performance compared to the analogous diblock copolymers. A series of predominantly (meth)acrylic comonomers are screened to identify optimal surface activity for foam stabilization of aqueous ethanol solutions. In particular, all-acrylic statistical copolymers comprising trimethylhexyl acrylate and poly(ethylene glycol) acrylate, P(TMHA-*stat*-PEGA), confer strong foamability and also lower the surface tension of a range of ethanol–water mixtures to a greater extent than the analogous block copolymers. For ethanol-rich hand sanitizer formulations, foam stabilization is normally achieved using environmentally persistent silicone-based copolymers or fluorinated surfactants. Herein, the best-performing fully hydrocarbon-based copolymer surfactants effectively stabilize ethanol-rich foams by a mechanism that resembles that of naturally-occurring proteins. This ability to reduce the surface tension of low-surface-energy liquids suggests a wide range of potential commercial applications.

## Introduction

Polymeric surfactants are important for many industrial sectors, including detergents for home and personal care, printing inks, oil recovery, latex syntheses via emulsion polymerization, cosmetics, and as excipients for drug formulations.^1,2^ Polymeric surfactants can offer significant advantages over their small-molecule counterparts, including very low critical micellar concentrations (CMC), fine control over the hydrophile–lipophile balance (HLB), and enhanced emulsion stability.^3^ Block copolymer architectures in which hydrophilic and hydrophobic comonomers are spatially segregated into two or more blocks are particularly common. For example, Pluronics comprising distinct blocks of poly(ethylene oxide) (PEO) and poly(propylene oxide) (PPO) have found widespread use in many of the above industrial applications.^4,5^

Traditionally, the synthesis of block copolymer surfactants requires multiple steps and stringent reaction conditions using techniques such as anionic polymerization.^6^ However, the development of pseudo-living radical polymerization techniques in the 1990s has allowed the preparation of many functional block copolymers using less synthetically demanding conditions.^7,8^ Nevertheless, the specialty reagents that are required for such syntheses are typically expensive and toxic. In principle, this problem can be mitigated by targeting longer copolymer chains. Unfortunately, high molecular weight block copolymer surfactants (>15 kg mol^–1^) usually lead to lower surface activity.^9−12^ Thus, linear (multi)block copolymer architectures may not be optimal for polymeric surfactants.^13^ Amphiphilic copolymeric surfactants with branched, star-like, or dendritic architectures can also exhibit high surface activity.^1^ However, such architectures typically involve much more complex synthetic protocols and require reagents that are not readily applicable to industrial-scale synthesis.

Nature often employs proteins as polymeric surfactants, with amphiphilic character conferred by the hydrophilic and hydrophobic amino acid side-chains that decorate the polypeptide backbone.^14^ Stochastic evolutionary design has produced highly surface-active proteins that can reduce the surface tension of water to 25 mN m^–1^,^15^ which is rarely achieved when using synthetic hydrocarbon-based surfactants. The surface activity of certain proteins is exploited to prepare ultrastable foams. For example, the African tree frog creates robust protein-stabilized foams that can survive harsh environmental conditions and hence protect its spawn.^14,16,17^ Protein-based surfactants can be used for fire-retardant foams^18^ and also in the food and beverage industry for the stabilization of edible emulsions and foams.^19,20^

Notwithstanding the precise secondary and tertiary structures often formed by amphiphilic proteins, the distribution of hydrophilic and hydrophobic amino acids along polypeptide backbones resembles that of a statistical (or random) synthetic copolymer. Amphiphilic statistical copolymers have already shown considerable promise as emulsifiers.^21−24^ However, numerous reports suggest that amphiphilic statistical copolymers are inferior to their block copolymer analogues in terms of surface activity.^25−29^ Recently, the self-assembly of amphiphilic statistical copolymers^30−33^ and alternating copolymers^34^ has been explored but the precise relationship between chemical structure, the formation of colloidal aggregates in aqueous solution, and surface activity remains unclear. However, there is little doubt that the synthesis of such copolymers should be amenable to industrial scale-up.

Herein, we evaluate a library of amphiphilic hydrocarbon-based copolymers in terms of their ability to stabilize foams generated from various ethanol–water mixtures. By screening a wide range of hydrophilic/hydrophobic comonomer pairs and targeting various copolymer compositions, we identify new copolymer surfactants that can stabilize foams for ethanol-rich solutions required for hand sanitizer formulations. Foam stabilization experiments and surface tensiometry measurements demonstrate that such statistical copolymers are more surface-active than the analogous diblock copolymers. Furthermore, small-angle X-ray scattering (SAXS) studies indicate that statistical copolymers undergo unimolecular self-assembly in ethanol–water mixtures, implying an unfolding mechanism at the air–water interface that resembles that of natural protein surfactants. New environmentally-friendly foam stabilizers for ethanol–water mixtures are particularly timely given the unprecedented growth in the use of alcoholic hand sanitizer formulations during the global COVID-19 pandemic.^35^ In this context, such foams enable the convenient dispensing of an optimum ethanol dose for killing bacteria and viruses, whereas the use of gels or liquids typically results in wasteful overdosing and poor hand feel (stickiness).^36^ To ensure lethality toward bacteria and viruses, the ethanol content in such formulations should be 60–95% ethanol by volume (i.e., ca. 54–94% by mass).^35,37^ However, such ethanol–water mixtures exhibit relatively low surface tension (<28 mN m^–1^) and normally require silicone-based polymeric surfactants for foam stabilization. Such surfactants are considered problematic because of their poor ecotoxicological profile^38^ and their degradation into toxic byproducts.^39^ The alternative hydrocarbon-based surfactants identified herein are expected to offer environmental advantages in various commercial applications.

## Results and Discussion

### Screening Binary Combinations of Hydrophilic and Hydrophobic Comonomers

**Scheme 1:**
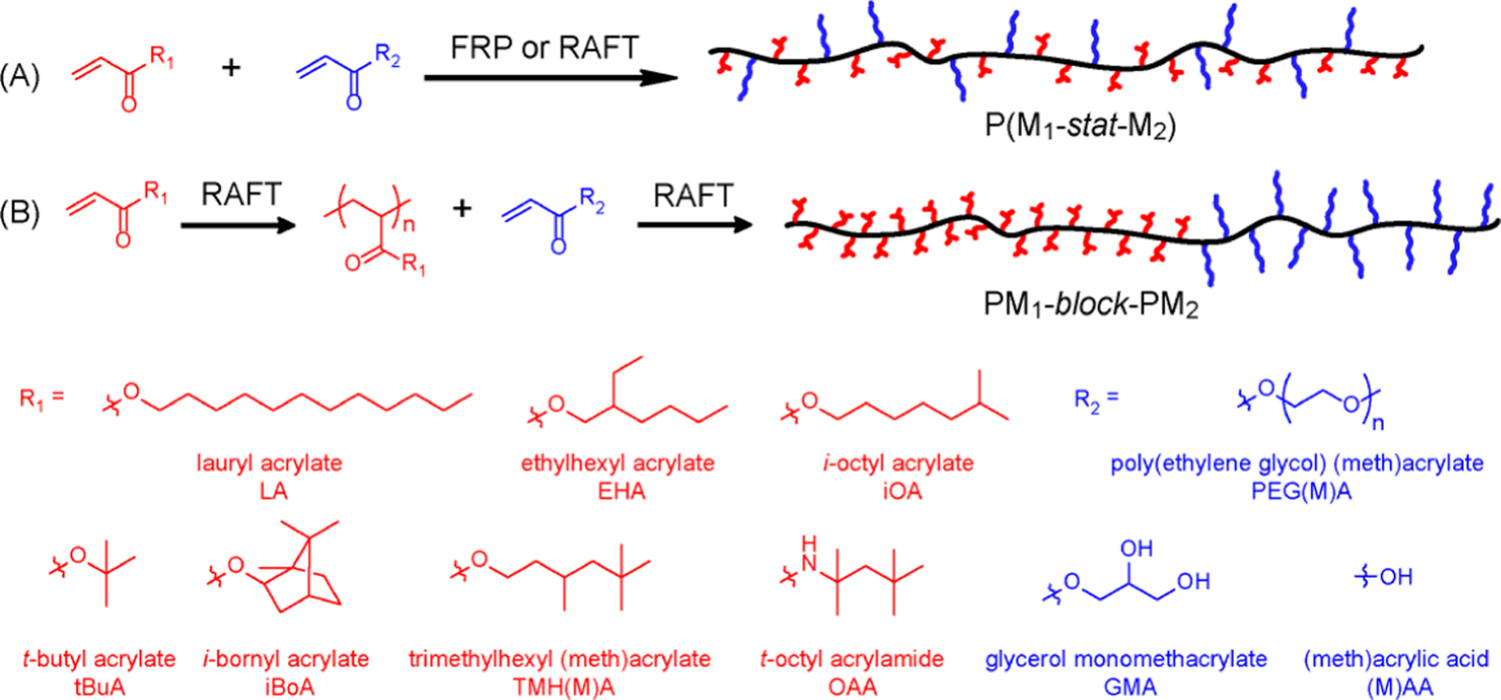
Synthetic Scheme Showing the Routes to Hydrocarbon-Based Amphiphilic Copolymer Surfactants Employed in This Study, Along with the Chemical Structures of the Various Hydrophobic (Red) and Hydrophilic (Blue) Comonomers from the Acrylate, Methacrylate, or Acrylamide Monomer Classes

During the synthesis of amphiphilic statistical copolymers via free-radical copolymerization in ethanol–water mixtures, we observed that the resulting reaction mixtures were prone to foaming when subjected to manual agitation (hand shaking), with similar surface activity being observed in the presence of organic solvents such as THF during work-up and purification. This serendipitous discovery led us to investigate the surfactant properties of amphiphilic statistical copolymers in binary ethanol–water mixtures relevant to hand sanitizer formulations. Conventional free-radical polymerization (FRP) is particularly well-suited for the synthesis of statistical copolymers using a wide range of (meth)acrylic and (meth)acrylamide monomers. Their broad commercial availability means that such monomers are applicable to high-throughput syntheses to generate libraries of amphiphilic copolymer surfactants.^40^ Accordingly, we performed a series of FRP syntheses employing numerous combinations of hydrophilic and hydrophobic comonomers (Scheme 1 and Table 1).

**Table 1:**
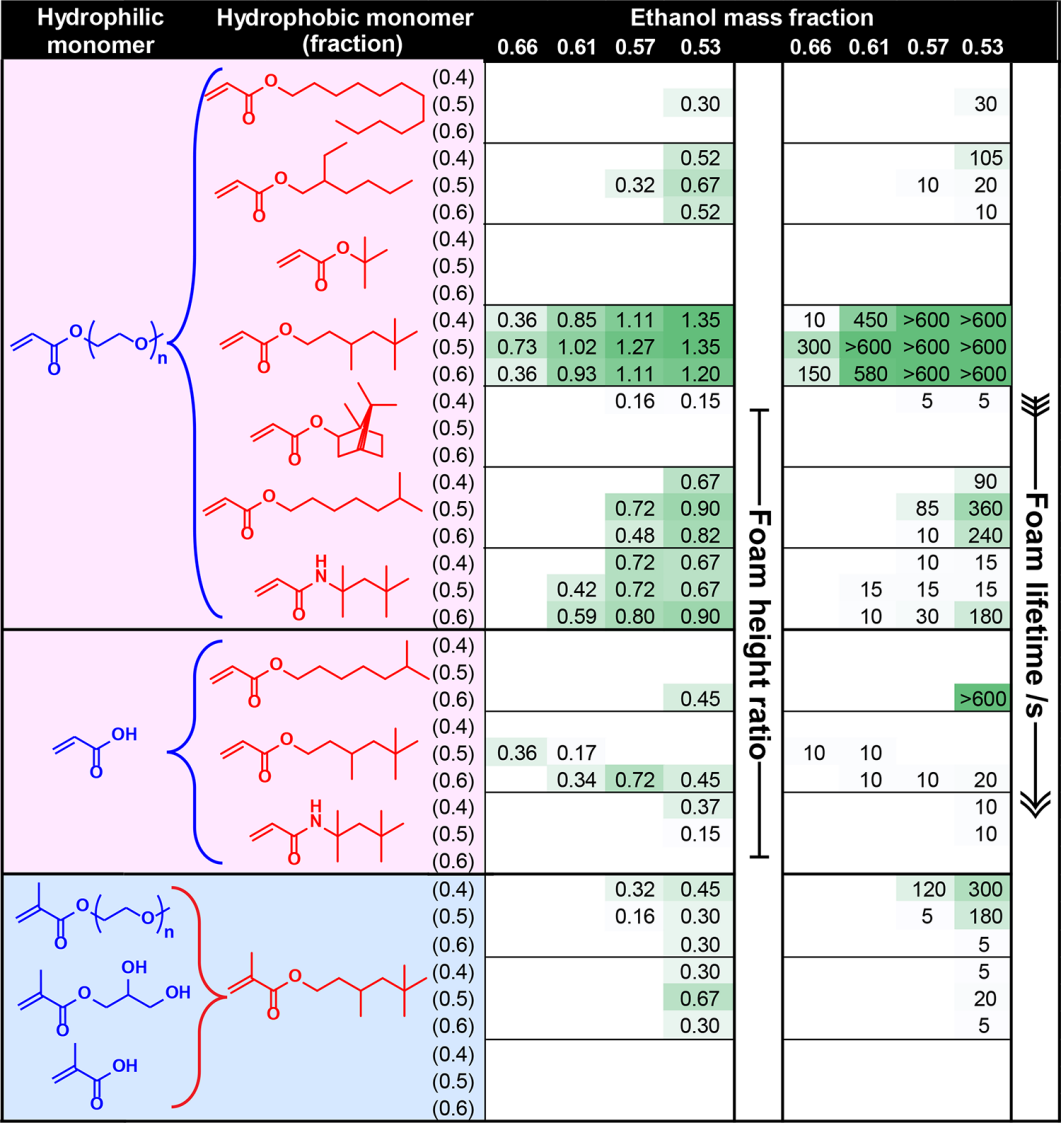
Summary of Foam Stabilization Performance for Various Ethanol-Water Mixtures Using a Library of Statistical Copolymer Surfactants Prepared by Free-Radical Copolymerization^a^

a [N.B. Green shading is used to denote more surface-active formulations].

We screened hydrophobic comonomers with differing alkyl side-chains and degrees of branching because such structural features are known to influence the surface activity of small-molecule surfactants.^41^ Meanwhile, hydrophilic components were selected from readily available non-ionic or anionic comonomers. With the exception of a single acrylate–acrylamide copolymer, syntheses were performed using all-acrylic (or all-methacrylic) formulations to ensure comparable comonomer reactivities and hence the formation of statistical (rather than blocky or gradient) copolymers.^32^ Comonomer feed mass fractions ranging from 0.40 to 0.60 were targeted to generate a range of HLB values, with ^1^H NMR analysis confirming that the final copolymer compositions were close to those used in the feed (Figure S1 and Table S1). The resulting copolymers are denoted using the convention ***M***_***x***_-***A***-***N***, where ***M*** and ***N*** refer to the hydrophobic and hydrophilic monomers, respectively, ***x*** is the mass fraction of the hydrophobic comonomer used in the feed, and ***A*** is the copolymer architecture (either statistical [*stat*] or *block*).

To assess the surface activity of each surfactant in various ethanol–water mixtures, we designed a high-throughput assay that required minimal copolymer. This protocol involved dissolving each copolymer in pure ethanol at 0.5% w/v prior to the successive addition of known aliquots of ultrapure water, with foamability being assessed by vortex agitation at each ethanol–water composition. Foam stabilization was initially examined in pure ethanol (γ_solvent/air_ = 22.3 mN m^–1^) and then for a series of ethanol–water mixtures containing 11, 20, 27, 34, 39, or 47% w/w water (at the highest water content γ_solvent/air_ = 29.0 mN m^–1^).^42^ Although the copolymer concentration necessarily decreases across this series, stronger foaming was typically observed at higher water contents (which correspond to higher γ_solvent/air_ values). The foam height ratio was used as a proxy for foamability, while foam lifetime was used as a metric for foam stability (see the Experimental section). Representative digital photographs of foams generated using the best-performing statistical copolymer surfactant are shown in Figure S2, alongside other copolymer surfactant foams for comparison.

The foam stabilization assay enables highly surface-active formulations to be rapidly identified, thus circumventing the need for laborious surface tension measurements on more than 30 copolymers. This assay also enables the comparison of these new statistical copolymer surfactants with various commercially available hydrocarbon-based surfactants such as Triton X-100, sodium dodecyl sulfate, and Pluronic F127. The three commercial surfactants did not produce stable foams for any of the ethanol–water mixtures investigated in this study. Thus, any statistical copolymer surfactant producing a stable foam under such conditions clearly exhibits strong surface activity well beyond that of typical hydrocarbon-based surfactants. In this context, we note that silicone-based block copolymers are typically used to generate foams from the ethanol-rich ethanol–water mixtures required for effective hand sanitizer formulations.^43^

A summary of the initial foaming assay data obtained for statistical copolymer surfactants is presented in Table 1. Entries are shaded using a color scale to indicate enhanced foamability (or foam height ratio, *F*) and foam lifetime (*F*_t_, seconds). Clearly, the nature of the hydrophilic and hydrophobic acrylic comonomers has a significant influence on the foam stabilization performance of these copolymer surfactants in various ethanol–water compositions. For example, the P(^t^BuA-*stat*-PEGA) copolymer produced no discernible foam for any binary solvent mixture, while P(LA-*stat*-PEGA), P(^i^OA-*stat*-AA), and P(OAA-*stat*-AA) copolymers only produced foams at the highest water content (47% w/w). The remaining copolymer surfactants can be ranked according to their relative foamability and foam stability performance across the range of ethanol–water mixtures as follows: P(TMHA-*stat*-PEGA) > P(OAA-*stat*-PEGA) > P(TMHA-*stat*-AA) > P(^i^OA-*stat*-PEGA) > P(EHA-*stat*-PEGA) > P(^i^BoA-*stat*-PEGA).

P(TMHA-*stat*-PEGA) copolymers significantly outperformed all other surfactants in terms of their foamability and foam stability. Thus, we hypothesized that using TMHMA, which is the methacrylic analogue of TMHA, should lead to similar performance for the analogous all-methacrylic statistical copolymer surfactants. Accordingly, P(TMHMA-*stat*-PEGMA), P(TMHMA-*stat*-GMA), and P(TMHMA-*stat*-MAA) were prepared targeting the same copolymer compositions and evaluated using the same foaming assay (see blue entries in Table 1). Surprisingly, each of these copolymers exhibited significantly lower foamability than the equivalent all-acrylic copolymer. For example, the P(TMHMA-*stat*-GMA) and P(TMHMA-*stat*-PEGMA) copolymers only displayed foamability at higher water contents (43–47% w/w). We will revisit these unexpected observations later.

Having observed inferior foamability for AA- or MAA-based surfactants relative to neutral PEGA-based copolymers, we sought to understand how surface activity varied with solution pH and ionic strength. Thus, an additional series of foaming assays was performed for P(TMHA_0.5_-*stat*-PEGA), P(TMHA_0.5_-*stat*-AA), and P(TMHMA_0.5_-*stat*-MAA) in which the aqueous component of the ethanol–water mixture comprised either 1.0 M NaCl or was adjusted to either pH 3 or pH 10 (using HCl or NaOH, respectively). Compared to the assay involving ultrapure water (Table 1), the foam stability of P(TMHA_0.5_-*stat*-PEGA) was marginally reduced when adjusting the aqueous component pH to pH 10 but remained essentially unchanged in the presence of 1.0 M NaCl or after adjusting the aqueous component pH to pH 3 (Table S2). On the other hand, the foamability conferred by P(TMHA_0.5_-*stat*-AA) was marginally reduced in the presence of 1.0 M NaCl or at pH 3 but remained essentially unchanged at pH 10. Finally, P(TMHMA_0.5_-*stat*-MAA) could only stabilize foam in the presence of 1.0 M NaCl. Overall, the poor foamability of copolymers containing ionizable AA or MAA repeat units was not significantly enhanced whether these moieties are fully protonated (pH 3), fully deprotonated (pH 10), or charge-screened (1.0 M NaCl). This suggests that the surface activity is not significantly affected by the degree of ionization of the carboxylic acid repeat units under these conditions. Moreover, P(TMHA_0.5_-*stat*-PEGA) remained highly surface-active and was able to stabilize foams in the presence of each type of modified aqueous phase (Table S2). Furthermore, similar foaming performance was obtained even after the deliberate addition of up to 8% comonomer by mass, which suggests that foaming is not caused by small-molecule impurities (Table S3). The non-ionic nature of this copolymer surfactant ensures good performance over a range of conditions, which is likely to be an advantage for many industrial applications. Finally, P(TMHA-*stat*-PEGA) copolymers were also prepared containing TMHA mass fractions of either 0.25 or 0.75 but in each case, the copolymer foamability was significantly poorer than that observed for the formulations shown in Table 1.

Each of the statistical copolymers presented in Table 1 was prepared by FRP, as indicated by their relatively high dispersities (*Đ* = 1.9–2.4, see Table S1). Further copolymers [hereinafter denoted as **R**-P(TMHA_0.4-0.6_-*stat*-PEGA)] were prepared using reversible addition–fragmentation chain transfer (RAFT) copolymerization^7^ to understand the influence of chain length distribution on foam stabilization performance. GPC analysis of RAFT-synthesized copolymers confirmed that they possessed significantly narrower molecular weight distributions relative to FRP-synthesized copolymers (Figure S3A). Foam stabilization assays using these lower dispersity copolymers indicated similar foamability and foam stability as that obtained using P(TMHA_0.4-0.6_-*stat*-PEGA) (see Table 2). This suggests that foamability is insensitive to the copolymer molecular weight distribution under these conditions. RAFT polymerization also provides good control over the copolymer *M*_n_, thus enabling the influence of chain length on foam stabilization to be investigated. Thus, three R-P(TMHA_0.5_-*stat*-PEGA) copolymers were prepared with *M*_n_ values of 4, 8, or 12 kg mol^–1^ by adjusting the comonomer/RAFT agent molar ratio (see Table S1 and Figure S3B). Each copolymer was subjected to the same foaming assay, and comparable foamability and foam stability were observed in all three cases (Table 2). However, the 4 kg mol^–1^ copolymer exhibited marginally lower foamability for all ethanol–water compositions investigated, suggesting slightly lower surface activity. Clearly, using RAFT polymerization to gain better control over the copolymer *M*_n_ and produce narrower molecular weight distributions does not lead to superior performance for P(TMHA-*stat*-PEGA) copolymers in foam stabilization experiments.

**Table 2:**
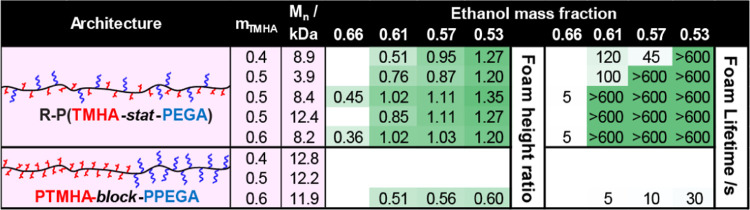
Summary of the Foam Stabilization Performance Observed for a Library of Statistical and Block Copolymer Surfactants Prepared by RAFT Solution Polymerization

There are relatively few studies of the effect of copolymer architecture on surface activity that explicitly compare statistical and block copolymer architectures.^25−28^ Herein, we prepared diblock copolymer analogues (i.e., PTMHA_0.4-0.6_-*block*-PPEGA) of the most surface-active statistical copolymers P(TMHA_0.4-0.6_-*stat*-PEGA) using RAFT polymerization.^7,8^ Sequential monomer addition enabled the production of well-defined diblock copolymers with *M*_n_ values of 11.8–12.9 kg mol^–1^ and relatively narrow molecular weight distributions (*Đ* = 1.2–1.3), as judged by GPC analysis (see Figure S4 and Table S1). A relatively low copolymer M_n_ was targeted in view of observations made by Matsuoka et al., who found that diblock copolymers with *M*_n_ > 15 kg mol^–1^ became surface-inactive.^11,12^ Furthermore, PTMHA_0.4_-*block*-PPEGA, PTMHA_0.5_-*block*-PPEGA, and PTMHA_0.6_-*block*-PPEGA exhibited comparable GPC *M*_n_ values to those obtained for the polydisperse statistical copolymers prepared by conventional FRP (*M*_n_ = 11.5–13.2 kg mol^–1^). Nevertheless, foam stabilization assays conducted in ethanol–water mixtures using PTMHA_0.4-0.6_-*block*-PPEGA indicated very poor foamability for all copolymer compositions compared to P(TMHA_0.4-0.6_-*stat*-PEGA). Foamability was only observed for PTMHA_0.6_-*block*-PPEGA for the most water-rich compositions (see Table 2), while foam heights and lifetimes were significantly lower than those observed for the equivalent statistical copolymer.

### Surface Tension Analysis

According to the foamability assays, several statistical copolymer surfactants can stabilize long-lasting foams in low surface energy solvents (<29 mN m^–1^). Notably, the analogous diblock copolymers, and also a range of commercial surfactants, do not perform as effectively under such conditions. This suggests that these statistical copolymer surfactants are more surface-active than many other hydrocarbon-based surfactants. In several prior studies, tensiometry was used to show that block copolymers reduced the interfacial tension (γ_solvent/air_) to a greater extent than statistical/random copolymers, thus indicating superior surfactant performance.^25−27^

**Figure 1:**
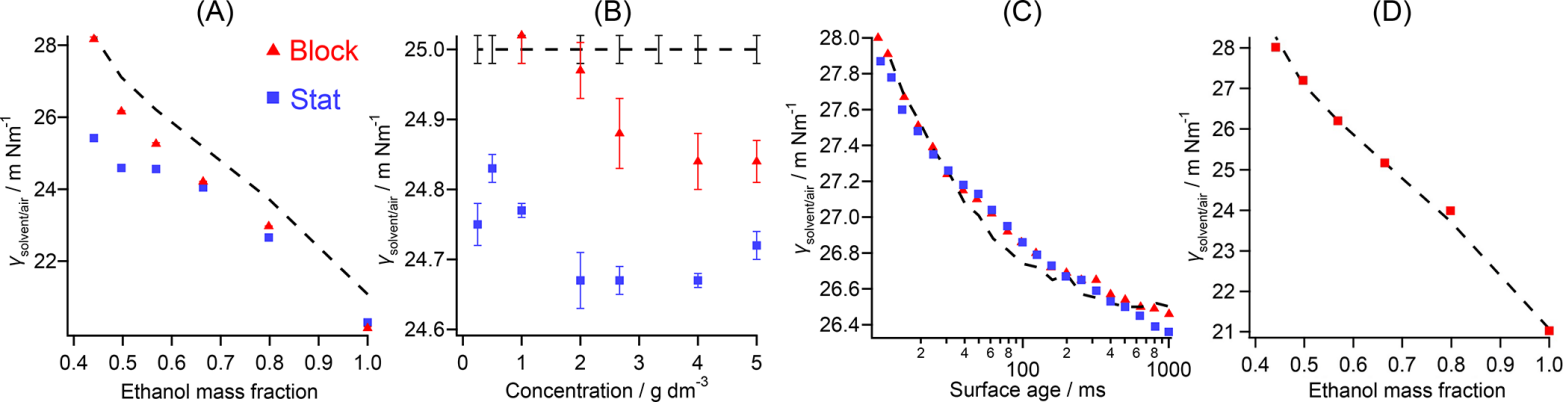
Surface tension measurements performed using various ethanol−water binary solutions of P(TMHA_0.5_-*stat*-PEGA) (blue squares) and PTMHA_0.5_-*block*-PPEGA (red triangles). (A) Variation in surface tension with ethanol–water composition, relative to the pure binary solvent mixtures (dashed line). Error bars calculated from standard deviations are smaller than the symbols in all cases. (B) Surface tension vs concentration plot for copolymers dissolved in 61.2% w/w ethanol. Error bars calculated from standard deviations are shown. (C) Bubble pressure tensiometry (BPT) analysis of copolymer surfactants at 4.0 g dm^–3^ in 61.2% w/w ethanol. (D) Surface tension measurements recorded for a range of ethanol–water mixtures containing P(TMHMA_0.5_-*stat*-PEGMA) compared to the surface tension data obtained for each corresponding binary solvent mixture alone (black dashed line).

To correlate performance in the foamability assay with surface activity, we conducted a series of ring tensiometry measurements using P(TMHA_0.5_-*stat*-PEGA) and PTMHA_0.5_-*block*-PPEGA in various ethanol–water mixtures (Figure 1A). Copolymer stock solutions were prepared at 5.0 g dm^–3^ in ethanol and γ_solvent/air_ was determined at 25 °C in various ethanol–water mixtures prepared by serial dilution with water to replicate the foaming assay conditions.

At the highest ethanol mass fractions (79–100% w/w), γ_solvent/air_ was only marginally lowered relative to the pure binary solvent mixtures for each copolymer (Figure 1A). However, at 57% w/w ethanol (for which we measured γ_solvent/air_ = 26.21 ± 0.03 mN m^–1^), the statistical copolymer lowered the surface tension to 24.56 ± 0.01 mN m^–1^, whereas the diblock copolymer only led to a reduction to 25.26 ± 0.03 mN m^–1^. This observation is consistent with the foaming assay: only the statistical copolymer was able to produce a stable foam at this ethanol–water composition. The difference in γ_solvent/air_ between the statistical and diblock copolymers gradually increased with addition of further water. At 44% w/w ethanol (not studied in the foamability assay), PTMHA_0.5_-*block*-PPEGA produced the same γ_solvent/air_ as that for the pure solvent mixture (ca. 28.2 mN m^–1^). This implies that these copolymer chains no longer occupied the interface under such conditions.

Surface tensiometry measurements were also conducted for copolymer concentrations of 0.1–5.0 g dm^–3^ to identify the critical micellar concentration (CMC) for P(TMHA_0.5_-*stat*-PEGA) and PTMHA_0.5_-*block*-PPEGA in an ethanol–water composition containing 61.2% w/w ethanol (Figure 1B). The diblock copolymer reduced γ_solvent/air_ between 2.7 and 5.0 g dm^–3^, but for lower copolymer concentrations, γ_solvent/air_ was equivalent to that of the pure solvent mixture (25.00 ± 0.02 mN m^–1^). These two distinct regimes suggest that the diblock copolymer exhibits a CMC between 2.0 and 2.7 g dm^–3^, while above this concentration, the solvent–air interface is saturated with copolymer chains and micelles are present in the bulk solution. Notably, the statistical copolymer reduced γ_solvent/air_ by ca. 0.1–0.2 mN m^–1^ more than the diblock copolymer at every concentration investigated. Moreover, the former copolymer exhibits no CMC over this concentration range and remained surface-active even at 0.1 g dm^–3^.

Bubble pressure tensiometry (BPT) measurements performed at 4.0 g dm^–3^ in the presence of 61.2% w/w ethanol (Figure 1C) enabled comparison of the dynamic interfacial behavior of these surfactants. Statistical copolymer surfactants produced a reduction in γ_solvent/air_ even for short surface aging times (<30 ms) and maintained relatively low γ_solvent/air_ values at longer surface aging times (>500 ms). These data suggest that statistical copolymer surfactants adsorb much faster at the solvent/air interface than block copolymer surfactants. To understand how the copolymer morphology in solution influences the chain dynamics, we studied various copolymer solutions using SAXS.

### Bulk Solution and Interfacial Self-assembly

The consistently larger reduction in γ_solvent/air_ by P(TMHA_0.5_-*stat*-PEGA) indicates that these copolymer chains adsorb more strongly at the interface than the analogous diblock copolymer chains. Establishing a sufficient surface tension gradient at the interface is a prerequisite for foam stabilization^44^ and accounts for the enhanced performance of the statistical copolymer in ethanol–water mixtures. The self-assembly of amphiphilic copolymers in solution is intrinsically linked to their surface activity. In aqueous media, block copolymers self-assemble to form multimolecular micelles with hydrophilic blocks comprising the shell and hydrophobic blocks within the core,^45^ and surface activity is higher above the CMC. The size and shape of these micelles depend mainly on the overall copolymer molecular weight and the relative volume fractions of the two blocks.^46^ On the other hand, the self-assembly of amphiphilic statistical copolymers in selective solvents is less well understood.^30−33,47^ The presence of hydrophilic and hydrophobic repeat units within the same copolymer chain results in intramolecular chain-folding such that hydrophilic pendent groups are presented at the copolymer/solvent interface, while hydrophobic groups remain buried within the cores. Importantly, this can lead to the formation of micelles with relatively low aggregation numbers; indeed, unimolecular aggregates can be obtained under certain conditions.^31,33^ Clearly, these two distinct self-assembly mechanisms must influence the propensity of copolymer chains to populate an interface within a given timescale.

**Figure 2:**
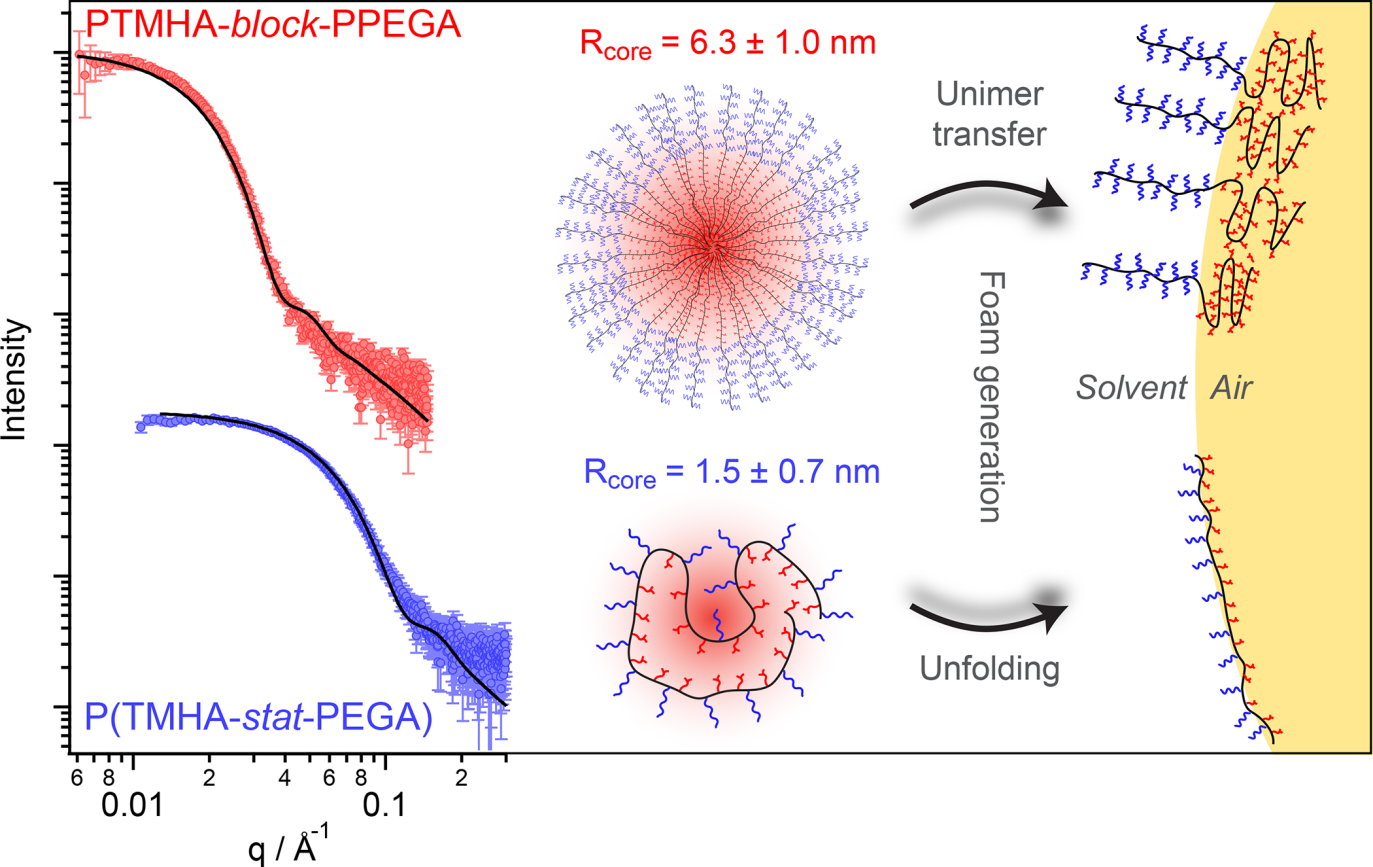
One-dimensional (1D) SAXS patterns recorded for P(TMHA_0.5_-*stat*-PEGA) (blue) and PTMHA_0.5_-*block*-PPEGA (red) at 3.0% w/w in an ethanol–water mixture containing 61.2% w/w ethanol at 22 °C. Data fits obtained using a spherical micelle model are shown as black lines. The corresponding schematic depicts a multimolecular micelle formed by the diblock copolymer and a unimolecular micelle formed by the statistical copolymer (not to scale) and their proposed conformations when adsorbed at the solution–air interface (right).

We studied the self-assembly behavior of the P(TMHA_0.5_-*stat*-PEGA) and PTMHA_0.5_-*block*-PPEGA copolymers above the CMC of the latter (3.0% w/w) and at an intermediate ethanol fraction (61.2% w/w). Under such conditions, only the statistical copolymer formed a stable foam. SAXS was used to characterize the micelles that are formed in solution (Figure 2). The presence of intensity minima (*q*_min_) in both scattering patterns indicated the presence of nanoparticles, while the low *q* gradient of approximately zero indicated a spherical morphology in both cases. The significantly higher *q*_min_ observed for P(TMHA_0.5_-*stat*-PEGA) compared to PTMHA_0.5_-*block*-PPEGA suggests significantly different self-assembly behavior.

A satisfactory fit to the SAXS pattern recorded for the former copolymer was obtained using a well-known spherical micelle model^48^ by fitting the core radius based on an individual hydrophobic repeat unit and the shell *R*_g_ value based on the expected *R*_g_ of the PEG side-chain^49^ (see the Supporting Information for model parameters). It is assumed that the micelle shell mainly comprises solvated PEG side chains with low X-ray contrast, thus scattering is dominated by the hydrophobic TMHA-based micelle core. This SAXS model indicated the presence of spherical micelles with a mean core radius, *R*, of 1.5 ± 0.7 nm, which is consistent with the size of unimolecular micelles formed by other amphiphilic statistical copolymers reported in the literature.^31,33^ The aggregation number (*N*_agg_) could be estimated by comparing the micellar volume measured by SAXS with the expected volume occupied by a single copolymer chain. Using the *M*_n_ value determined by GPC, an *N*_agg_ of 1.18 was calculated (see the Supporting Information). The use of poly(methyl methacrylate) calibration standards for the GPC measurements incurs a systematic error in *M*_n_, which most likely accounts for the modest deviation of *N*_agg_ from unity. Thus, these data suggest that P(TMHA_0.5_-*stat*-PEGA) forms unimolecular micelles in ethanol–water mixtures. Using the same spherical micelle model, but with the core radius based on a PTMHA block, PTMHA_0.5_-*block*-PPEGA micelles were estimated to have a core radius of 6.3 ± 1.0 nm and an *N*_agg_ of 92, which are physically reasonable values for such an amphiphilic diblock copolymer.^50^

To produce an air-in-liquid foam, sufficient surfactant must adsorb to create a surface tension gradient and stabilize the new interfacial area that is generated. For conventional surfactants, micelles must first dissociate to form individual surfactant molecules and the latter species then diffuse to the solvent–air interface. Such micelle dissociation events introduce a relatively high energetic penalty.^51^ Alternatively, the relatively large diblock copolymer micelles could diffuse slowly to the interface and undergo adsorption-induced dissociation. Once adsorbed, the hydrophobic tails are oriented toward the gas phase, while hydrophilic head-groups remain solvated within the near-surface of the interface. However, the foam stabilization mechanism for the statistical copolymer surfactants reported herein involves a different mechanism. In this case, individual copolymer chains form micelles by chain folding such that the hydrophobic groups are located within the cores, while hydrophilic PEG groups form the solvated micelle shell (see the schematic cartoon shown in Figure 2). Such unimolecular micelles must undergo a conformational transition at the interface to enable the buried hydrophobic TMHA groups to interact with the gas phase, while the pendent PEG groups remain solvated in the aqueous milieu.

Compared to multimolecular micelles formed by the diblock copolymer, unimolecular micelles can rapidly diffuse to the interface. Moreover, while the former must first dissociate into unimers, statistical copolymer chains can simply undergo a conformational transition at the interface to present their hydrophobic groups (Figure 2). This rearrangement has an entropic penalty associated with the transition from collapsed and compact chains to stretched conformations. However, this is more than offset by the gain in favorable enthalpic interactions, which enables efficient foam stabilization even with relatively low energy input (e.g., manual hand shaking).

Further evidence for the unfolding mechanism of statistical copolymer surfactants is provided by considering their chain mobility, for which the copolymer *T*_g_ is a proxy. Differential scanning calorimetry (DSC) studies indicate a *T*_g_ of −64 °C for P(TMHA_0.5_-*stat*-PEGA) (see Figure S5A). Unfortunately, no *T*_g_ could be determined for the corresponding P(TMHMA_0.5_-*stat*-PEGMA) copolymer. However, DSC analysis of PTMHA and PTMHMA homopolymers confirmed a significantly higher *T*_g_ for the methacrylic analogue (Figure S5B). SAXS analysis confirms that P(TMHMA_0.5_-*stat*-PEGMA) also forms unimolecular micelles in ethanol–water mixtures (Figure S6) but surface tensiometry measurements indicate that this copolymer does not reduce the surface tension over the full range of ethanol–water mixtures studied (Figure 1D). Nevertheless, P(TMHMA-*stat*-PEGMA) copolymers were still able to stabilize foams at higher water contents, although foamability and foam stability were much lower than those observed for P(TMHA-*stat*-PEGA) copolymers (Table 1). We hypothesize that in the case of P(TMHMA_0.5_-*stat*-PEGMA), chains can only occupy the interface when a significant amount of energy is applied (i.e., vortex mixing) to drive the transition from compact unimolecular micelles to an extended conformation at the interface (as shown in Figure 2). Given the lower flexibility of the methacrylic backbone,^32^ this transition cannot occur under the static conditions used in tensiometry. Clearly, chain mobility is a critical parameter that significantly affects the performance of statistical copolymer surfactants. This important insight also explains why P(OAA-*stat*-PEGA), another copolymer containing a high *T*_g_ hydrophobic comonomer,^52^ is a much less effective foam stabilizer than P(TMHA_0.5_-*stat*-PEGA).

According to the surface tensiometry data, the trimethylhexyl (TMH) groups within statistical copolymers can adsorb more efficiently and more rapidly at the interface than those within the analogous diblock copolymer chains. Hence, statistical copolymers produced lower surface tensions at all concentrations and for most ethanol–water mixtures. For maximum surface coverage by PTMHA-*block*-PPEGA, the PTMHA chain should adsorb parallel to the interface with each of its TMH groups directed toward the air phase. However, such a conformation would incur a significant enthalpic penalty. It is much more likely that the PTMHA chain collapses to form a desolvated coil (Figure 2), hence a suboptimal proportion of TMHA groups is located at the interface. In contrast, the statistical copolymer chain can readily adopt an extended conformation such that the majority of its pendent hydrophobic TMH groups are adsorbed at the air phase, while its hydrophilic PEG groups remain solvated within the near-surface of the solution. This produces a significantly higher interfacial area per molecule for P(TMHA-*stat*-PEGA) compared to PTMHA-*block*-PPEGA and therefore enables stabilization of a greater foam volume.

Notably, similar micellar dimensions were observed for several other statistical copolymer surfactants examined in this study (e.g., P(TMHMA_0.5_-*stat*-PEGMA), P(LA_0.5_-*stat*-PEGA), P(^i^OA_0.5_-*stat*-PEGA), and R-P(TMHA_0.5_-*stat*-PEGA), Figure S6). This suggests that a wide range of non-ionic statistical copolymer surfactants can form unimolecular micelles. However, given the much lower foamability observed for P(TMHMA_0.5_-*stat*-PEGMA), P(LA_0.5_-*stat*-PEGA), and P(^i^OA_0.5_-*stat*-PEGA), such self-assembly behavior is not the sole prerequisite for interfacial activity. Empirically, we find that the most efficient surfactants contain hydrophobic comonomers comprising more than two methyl groups per repeat unit (i.e., TMHA, ^i^OA, and OAA). Prior studies of both hydrocarbon and silicone-based small-molecule surfactants indicate that more branched hydrophobic moieties lower the surface tension to a greater extent.^41,53^ This is believed to be related to weaker tail–tail interactions and the large volumes occupied by bulky hydrophobic tails at an interface.^54^ The length of the spacer group between the backbone and methyl groups also appears to be an important determinant of surface activity: copolymers bearing methyl-rich ^t^BuA repeat units cannot stabilize foams in ethanol–water mixtures (Table 1). Notably, our hydrocarbon-based surfactants compare favorably to perfluorinated surfactants as foam stabilizers for ethanol–water mixtures. More specifically, for a comparable surfactant concentration in 50% v/v ethanol, our optimized P(TMHA-*stat*-PEGA) copolymer surfactant reduced the surface tension to 25.4 mN m^–1^ (vs 28 mN m^–1^ for a perfluorinated surfactant).^55,56^

Moreover, this hydrocarbon surfactant also produced more stable foams. Interestingly, this unfolding mechanism resembles how some protein-stabilized foams are generated in nature:^57^ when subjected to mechanical stimulation (such as the “whipping” mechanism used by tree frogs^17^), globular proteins can unfold in solution to expose buried hydrophobic amino acids that stabilize the air–water interface to produce long-lasting foams. We also note that the molecular weight of these statistical copolymers is within the range determined for hydrophobins (≤20 kg mol^–1^^58^), which are highly surface-active proteins found in filamentous fungi.^59^ As far as we are aware, this mechanism of foam stabilization has not been previously reported for synthetic copolymers. In principle, scattering methods such as small-angle neutron scattering^60^ could provide further insight regarding the interfacial copolymer structure and foam stabilization mechanism.

## Conclusions

According to various studies, block copolymers are preferred to statistical copolymers for surfactant performance.^25−28^ Nevertheless, herein we show that judicious selection of pairs of acrylic comonomers leads to superior surface activity for statistical copolymers. The influence of various structural parameters (e.g., the chemical nature of the hydrophobic and hydrophilic comonomers, copolymer composition, *M*_n_, copolymer dispersity, and copolymer chain mobility) on the foam stabilization performance of a series of copolymer surfactants was examined. A foamability assay was employed to rapidly identify the most appropriate comonomer pairs, with the chemical nature of the hydrophobic comonomer found to strongly influence surface activity. The strong foamability exhibited by P(TMHA-*stat*-PEGA) copolymers suggests that the combination of a highly branched hydrophobic comonomer with a non-ionic hydrophilic comonomer is optimal.

The high surface activity demonstrated herein for certain statistical copolymers is related to their highly mobile all-acrylic backbones and their formation of unimolecular micelles in solution. This facilitates fast diffusion to the air–water interface and rapid conformational rearrangement of each copolymer chain to enable its hydrophobic comonomer units to interact with the air phase. In contrast, multimolecular block copolymer micelles must either dissociate into unimers that then diffuse to the surface or whole micelles must slowly diffuse to the air–water interface prior to micellar adsorption.^61^ Thus, amphiphilic statistical copolymers exhibit superior foam stabilization for a range of ethanol-rich ethanol–water mixtures that are relevant for hand sanitizer formulations. Indeed, statistical copolymer surfactants can stabilize high-quality foams when extruding hand sanitizer formulations through a model soap dispenser (see Video S1).

In nature, evolutionary design has produced various highly surface-active proteins comprising hydrophilic and hydrophobic amino acids distributed along polypeptide backbones in non-blocky sequences. Herein, we show that the informed design of new hydrocarbon-based amphiphilic statistical copolymers can also lead to remarkable surface activity. Further optimization of the synthetic protocol (e.g., monomer feeding protocols, temperatures, initiator systems) is expected to produce further performance enhancements that may even exceed that of natural proteins. Importantly, these synthetic copolymer surfactants offer both cost and environmental advantages over the fluorocarbon- and siloxane-based surfactants that are currently widely used in the chemical industry.

## Experimental Section

### Materials

Glyceryl monomethacrylate (GMA; 98%, supplied by GEO Specialty Chemicals), poly(ethylene glycol) methyl ether acrylate (PEGA, *M*_n_ = 480, Sigma-Aldrich), poly(ethylene glycol) methyl ether methacrylate (PEGMA, *M*_n_ = 950, Sigma-Aldrich), acrylic acid (AA, 99%; Fisher), methacrylic acid (MAA, ≥99%; Merck), lauryl acrylate (LA, >98%, TCI Chemicals), *iso*-octyl acrylate (^i^OA, >90%; Sigma-Aldrich), 2-ethylhexyl acrylate (EHA, Alfa Aesar, 98%), *iso*-bornyl acrylate (^i^BA 99%; Alfa Aesar), *tert*-butyl acrylate (^t^BuA, 99%; Alfa Aesar), 3,5,5-trimethylhexyl acrylate (TMHA; Sigma-Aldrich), 3,5,5-trimethylhexyl methacrylate (TMHMA, ABCR), and *tert*-octyl acrylamide (OAA; supplied by ABCR, Germany) were all used as received. Asoisobutyronitrile (AIBN) was recrystallized twice from methanol. Ethanol (99.8%, Fisher), methanol (>99%, Sigma-Aldrich), THF, and *n*-heptane (>99%, Sigma-Aldrich) were used as received. 2-(Dodecylthiocarbonothioylthio)-2-methylpropionic acid (DDMAT, 98%) was purchased from Sigma-Aldrich and used as received. MilliQ water was obtained from an Elga Elgastat Option 3A Water Purifier system.

### Synthesis of a P(TMHA-*stat*-PEGA) Statistical Copolymer by Free-Radical Copolymerization

AIBN (2.5 mg) was weighed into a 7 mL glass vial, followed by PEGA (0.25 g), TMHA (0.25 g), and ethanol (2.0 g). A magnetic flea was added to this vial, which was sealed using a rubber septum. After vortex mixing for 10 s to achieve a homogeneous solution, the reaction mixture was degassed on ice by sparging with nitrogen gas for 20 min. The vial was then immersed in an oil bath at 70 °C and the reaction mixture was stirred continuously for 4 h. After removing the vial from the oil bath, most of the solvent was evaporated under a gentle flow of nitrogen. The vial was then transferred to a vacuum oven at 70 °C to remove any residual solvent and unreacted monomer (the latter either by evaporation and/or reaction). The copolymer composition was determined by ^1^H NMR spectroscopy analysis in CDCl_3_ and the copolymer molecular weight and dispersity were assessed by GPC using a THF eluent. Following vacuum treatment, the residual comonomer was less than 2% in all cases.

### Synthesis of a Statistical Copolymer R-P(TMHA-*stat*-PEGA) by RAFT Solution Polymerization

AIBN (2.50 mg) and DDMAT (11 mg) were weighed into a 7 mL glass vial, followed by PEGA (0.25 g), TMHMA (0.25 g), and ethanol (2.0 g). A magnetic flea was added to this vial, which was sealed using a rubber septum. After vortex mixing for 10 s to achieve a homogeneous solution, the reaction mixture was degassed on ice by sparging with nitrogen gas for 20 min. The vial was then immersed in an oil bath at 70 °C and the reaction mixture was stirred for 24 h. After removing the vial from the oil bath, solvent and any unreacted monomer were removed overnight at 70 °C using a vacuum oven. The copolymer composition was determined by ^1^H NMR spectroscopy analysis in CDCl_3_ and its molecular weight and dispersity were assessed by GPC using a THF eluent. Following vacuum treatment, the residual monomer was less than 2 mol % in all cases.

### Synthesis of a PTMHA-*block*-PPEGA Diblock Copolymer by RAFT Solution Polymerization

AIBN (2.5 mg) and DDMAT (11 mg) were weighed into a 7 mL glass vial, followed by TMHA (0.25 g) and THF (0.50 mL). A magnetic flea was added to this vial, which was sealed using a rubber septum. After vortex mixing for 10 s to achieve a homogeneous solution, the reaction mixture was degassed on ice by sparging with nitrogen gas for 20 min. The vial was then immersed in an oil bath at 70 °C and the reaction mixture was stirred for 3 h. Then, the vial was removed from the oil bath, and an aliquot was extracted from the reaction solution for ^1^H NMR studies in CDCl_3_ and GPC analysis (THF eluent). The residual monomer was less than 5%. PEGA (0.25 g) and THF (0.80 mL) were then added to the vial and the mixture was degassed on ice by sparging with nitrogen gas for 20 min. The vial was then submerged in an oil bath at 70 °C and the reaction mixture was stirred for a further 10 h. After removing the vial from the oil bath, the solvent and any unreacted monomer were removed overnight at 70 °C using a vacuum oven. The mean composition of the diblock copolymer was determined by ^1^H NMR spectroscopy analysis in CDCl_3_ and its molecular weight and dispersity were assessed by GPC using THF eluent. Following vacuum treatment, the residual monomer was less than 2% in all cases.

### Analytical Techniques

Following copolymer dissolution in CDCl_3_, ^1^H NMR spectra were recorded using a Bruker AV1-400 MHz spectrometer with 64 scans being averaged per spectrum. Copolymer compositions were calculated in terms of the mass fraction of the hydrophobic comonomer (e.g., *m*_TMHA_) by comparing the integrals for the oxymethylene ester groups in the (meth)acrylic repeat units (eq 1), which were located at sufficiently different chemical shifts for the hydrophilic (e.g., *I*_PEGA_) and hydrophobic (e.g., *I*_TMHA_) monomers.
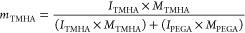
1GPC analysis was conducted in THF at 30 °C (containing 2.0% v/v triethylamine and 0.05% w/v 3,5-di-tert-4-butylhydroxytoluene) using a flow rate of 1.0 mL min^–1^. The GPC system comprised two Polymer Laboratories PL gel 5 μm Mixed C columns, an LC20AD ramped isocratic pump, and a WellChrom K-2301 refractive index detector operating at 950 ± 30 nm. Calibration was achieved using a series of near-monodisperse poly(methyl methacrylate) standards ranging from *M*_n_ = 1 280–330 000 g mol^–1^.

### Foamability Assay

After purification under vacuum, the dry copolymer (5.0 mg) was weighed into a 7 mL glass vial and dissolved in ethanol (1.00 mL). The only exceptions were P(OAA-*stat*-PEGA) and P(OAA-*stat*-AA), for which an aliquot of reaction solution (30 μL) following copolymerization was diluted with ethanol (970 μL). The ethanolic solution was agitated at 2850 rpm for 10 s using a vortex mixer (Cole-Palmer Vortex Mixer) and inspected for the appearance of bubbles. Ultrapure water, water adjusted to either pH 3 or 10, or 1 M NaCl, was then added in 0.10 mL aliquots, and the extent of foaming was evaluated by vigorous vortex mixing for 10 s. If the foam lifetime exceeded 5 s following vortex agitation, then the foam height (*h*) from the top of the liquid to the top of the foam was measured five times and the maximum height was recorded (excluding any particularly large bubbles). To compare data for different experiments, the foamability (*F*) was calculated using eq 2, where *V* is the initial volume of surfactant solution and *r* is the vial radius. Thus, the foamability, or foam ratio, is simply given by the foam volume normalized with respect to the original solution volume.
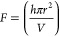
2Foam stability was arbitrarily defined as the time taken for the foam to break up to produce fewer than five bubbles at the surface of the liquid.

To test whether residual comonomers (<2% in all cases) had any effect on foamability, P(TMHA_0.5_-*stat*-PEGA) (which contained no detectable comonomer by ^1^H NMR spectroscopy) was deliberately contaminated with approximately 8% w/w PEGA and TMHA and then subjected to the same foaming assay. Essentially the same foamability and foam stability data were obtained within experimental error, suggesting that the surface activity of this copolymer was not affected by the residual comonomer.

### Surface Tensiometry

Surface tension measurements were performed using the Du Noüy ring method on an automated Lauda TD3 ring/plate tensiometer equipped with a 90:10 platinum/iridium ring (ring radius = 0.955 cm). Solvent mixtures or surfactant solutions (40 mL) were added to a glass beaker and at least 10 measurements were recorded for each sample at 25 °C until a standard deviation of less than 0.07 mN m^–1^ was obtained. For each copolymer, 200 mg was dissolved in ethanol (40 mL) and the surface tension of this solution was determined in the glass vessel. Following measurement, the copolymer solution was decanted into a jar, diluted with 8 mL water, and thoroughly mixed. The new solution (40 mL) was returned to the glass vessel for a second surface tension measurement (while maintaining the remaining solution in the jar). This protocol was repeated until the solution in the jar comprised 40 mL of water added to the initial 40 mL of ethanol (i.e., ethanol at 44.1% w/w). Concentration-dependent measurements were conducted by preparing an initial 5.0% w/w copolymer solution in an ethanol–water mixture containing 61.2 w/w % ethanol, which was sequentially diluted using the same ethanol–water mixture, as described above.

### Dynamic Surface Tensiometry

Measurements were conducted using a Krüss BP100 instrument using disposable polypropylene capillaries. The capillary radius was initially calibrated against ultrapure water. Dynamic surface tensions were determined on time scales ranging from 10 to 1000 ms and 10 measurements were averaged for time points. A thermostatted water bath was used to maintain the temperature at 20 °C. Dilute solutions were prepared by dissolving 4.0 g dm^–3^ copolymer in 50 mL of an ethanol/water mixture comprising 61.2% w/w ethanol. The copolymer was dissolved by vigorous stirring and each copolymer solution was allowed to equilibrate for at least 1 h prior to measurements.

### Small-Angle X-ray Scattering Analysis

SAXS analyses of copolymer dispersions were conducted using a Xeuss 2.0 (Xenocs) SAXS instrument equipped with a FOX 3D multilayered X-ray mirror. X-rays were generated from a liquid gallium MetalJet X-ray source (Excillum, λ = 1.34 Å) and collimated using two sets of scatterless slits. The scattering intensity was measured on a hybrid pixel area detector (Pilatus 1M, Dectris) at a sample-to-detector distance of approximately 1.20 m (calibrated using a silver behenate standard). Two-dimensional (2D) SAXS patterns were reduced to 1D plots by azimuthal integration using a Foxtrot software package.

### SAXS Data Modeling

Particle radii were calculated using a simple scattering model for spheres reported in the literature^30,31,48^ by employing the parameters summarized in Table S4. The mean aggregation number (*N*_agg_) was calculated from eq 3, i.e., from the ratio of the micelle core volume calculated from the particle radius measured by SAXS, *R*, to the theoretical micelle core volume, *V*_THMA_, assuming that no solvent was located within the micellar core (which is reasonable given that PTMHA is insoluble in ethanol).
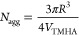
3*V*_THMA_ could be estimated from *V*_THMA_ = *M*_TMHA_/(*N*_A_ × ρ_THMA_). *M*_TMHA_ is the calculated molecular weight of the TMHA fraction of the copolymer, *M*_THMA_ = *M*_n_ × *m*_TMHA_, where *M*_n_ is the number-average molecular weight measured by THF GPC and *m*_TMHA_ is the TMHA mass fraction as measured by ^1^H NMR analysis. It was assumed that the density of the TMHA repeat units was equal to that of the monomer (ρ_THMA_ = 0.875 g cm^–3^) for both the block and statistical copolymers. Although this assumption is a likely source of error in such calculations, in practice the polydispersity of the statistical copolymer chains and error arising from GPC analysis against PMMA standards are more significant contributions to the deviation in *N*_agg_ from unity.

## Supporting Information Available

^1^H NMR spectra for selected copolymers, GPC curves for P(TMHA-*stat*-PEGA) copolymers, representative digital photographs for foams, additional foamability assay data, raw SAXS data, DSC curves, and a video showing foam generation within a model foam dispenser when using a “hand sanitizer”-type copolymer solution (PDF)
Video demonstrating that statistical copolymer surfactants can stabilize high-quality foams when extruding hand sanitizer formulations through a model soap dispenser (AVI)

## Supplementary Material

am2c09910_si_001.pdf

am2c09910_si_002.avi
