# Supplementary material for: Hydrocarbon-Based Statistical Copolymers Outperform Block Copolymers for Stabilization of Ethanol–Water Foams
Source: ACS Appl Mater Interfaces. 2022 Aug 19;14(34):39548–59. doi: 10.1021/acsami.2c09910 (PMC9437873; doi:10.1021/acsami.2c09910)
Supplement: Supplementary file 1 — am2c09910_si_001.pdf [file am2c09910_si_001.pdf]

Supporting Information

Hydrocarbon-based Statistical Copolymers Outperform Block Copolymers for Stabilization of Ethanol-Water Foams

James Jennings*†, Rebekah R. Webster-Aikman, Niall Ward-O'Brien, Andi Xie, Deborah L. Beattie, Oliver J. Deane, Steven P. Armes* and Anthony J. Ryan*

Dainton Building, Department of Chemistry, University of Sheffield,

Brook Hill, Sheffield, South Yorkshire, S3 7HF, UK.

*Authors to whom correspondence should be addressed

james.jennings@uni-graz.at; s.p.armes@sheffield.ac.uk; tony.ryan@sheffield.ac.uk

(A)

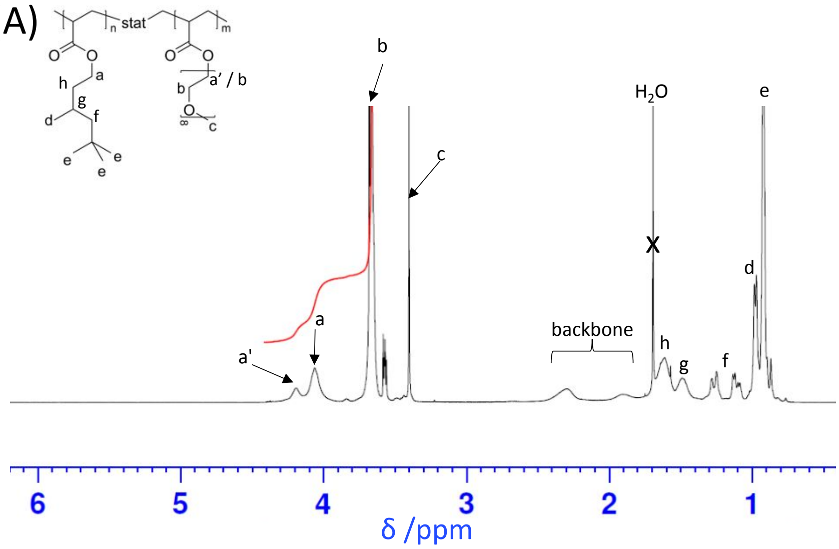
stat
a
h
g
d
f
e
e
e
a' / b
b
c
b
c
H_2O
e
X
d
backbone
h
g
f
a
a'
6
5
4
3
2
1
δ /ppm

(B)

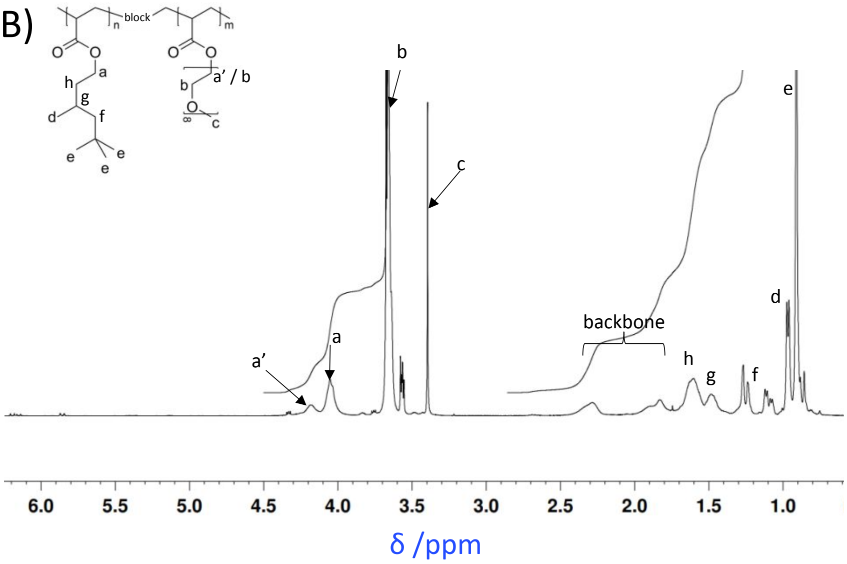
block
a
h
g
d
f
e
e
e
a' / b
b
c
b
c
e
d
backbone
h
g
f
a
a'
6.0
5.5
5.0
4.5
4.0
3.5
3.0
2.5
2.0
1.5
1.0
δ /ppm

(C)

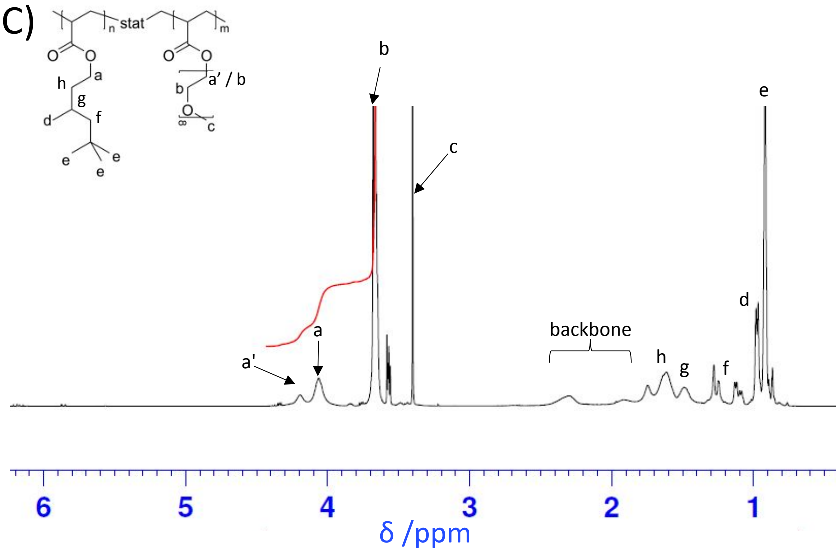
stat
a
h
g
d
f
e
e
e
a' / b
b
c
b
c
e
d
backbone
h
g
f
a
a'
6
5
4
3
2
1
δ /ppm

Figure S1. Representative ^{1}H NMR spectra ($CDCl_3$) recorded for (A) P($TMHA_{0.5}$-*stat*-PEGA), (B) $PTMHA_{0.5}$-*block*-PPEGA and (C) R-P($TMHA_{0.5}$-*stat*-PEGA).

Table S1. Summary of NMR and GPC data obtained for selected copolymers used in this study.

Copolymer	TMH(M)A feed mass fraction	TMH(M)A measured mass fraction[a]	GPC[b]	
			M_n	M_w/M_n
PTMHA-*block*-PPEGA	0.60	0.59	12.8	1.24
	0.50	0.51	12.2	1.21
	0.40	0.38	11.9	1.29
P(TMHA-*stat*-PEGA)	0.60	0.60	13.2	2.33
	0.50	0.50	12.6	2.14
	0.40	0.39	11.5	1.97
P(TMHMA-*stat*-PEGMA)	0.60	0.61	54.8	3.83
	0.50	0.49	29.6	2.74
	0.40	0.37	57.9	4.83
R-P(TMHA-*stat*-PEGA)	0.60	0.62	8.9	1.39
	0.50	0.52	3.9	1.22
	0.50	0.51	8.4	1.39
	0.50	0.49	12.4	1.64
	0.40	0.42	8.2	1.35

a. By ^{1}H NMR spectroscopy analysis.
b. In THF using a series of near-monodisperse PMMA calibration standards and a refractive index detector.

Table S2. Summary of the foam stabilization performance for various ethanol-aqueous mixtures when using three statistical copolymer surfactants synthesized by free radical polymerization. The aqueous solution either contained 1.0 M NaCl or was adjusted to either pH 3 or pH 10 in the absence of any added salt.

Hydrophile	Hydrophobe (water added)		F 0.66	F 0.61	F 0.57	F 0.53		Ft 0.66	Ft 0.61	Ft 0.57	Ft 0.53	
			**Ethanol mass fraction**					**Ethanol mass fraction**				
			**0.66**	**0.61**	**0.57**	**0.53**		**0.66**	**0.61**	**0.57**	**0.53**	
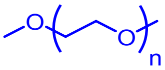	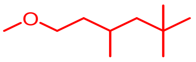	pH3	0.36	1.02	1.27	1.350	**Foamability**	5	360	600	600	**Foam Stability**
		pH10	0.00	0.93	1.35	1.35		0	210	300	600	
		NaCl	0.36	1.02	1.27	1.50		5	270	600	600	
—OH	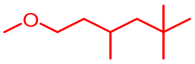	pH3		0.17				0	5			
		pH10	0.00	0.51	0.16			0	20	5		
		NaCl	0.00	0.00	0.00	0.00			0	0	0	
—OH	MA 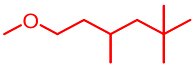	pH3										
		pH10										
		NaCl				0.45					5	

Table S3. Comparison of foam height ratio (F) and foam lifetime (Ft, in seconds) for P(TMHA$_{0.5}$-*stat*-PEGA) containing an undetectable level of comonomer contamination (as judged by ^{1}H NMR spectroscopy) with the same copolymer deliberately contaminated with approximately 8 % w/w comonomer.

Hydrophile	Condition	Ethanol mass fraction 0.66	0.61	0.57	0.53		Ethanol mass fraction 0.66	0.61	0.57	0.53	
	Purified polymer		0.93	1.03	1.20	**F**		270	450	600	**Ft**
	8 wt% monomers		0.93	0.95	1.20		0	270	450	600	

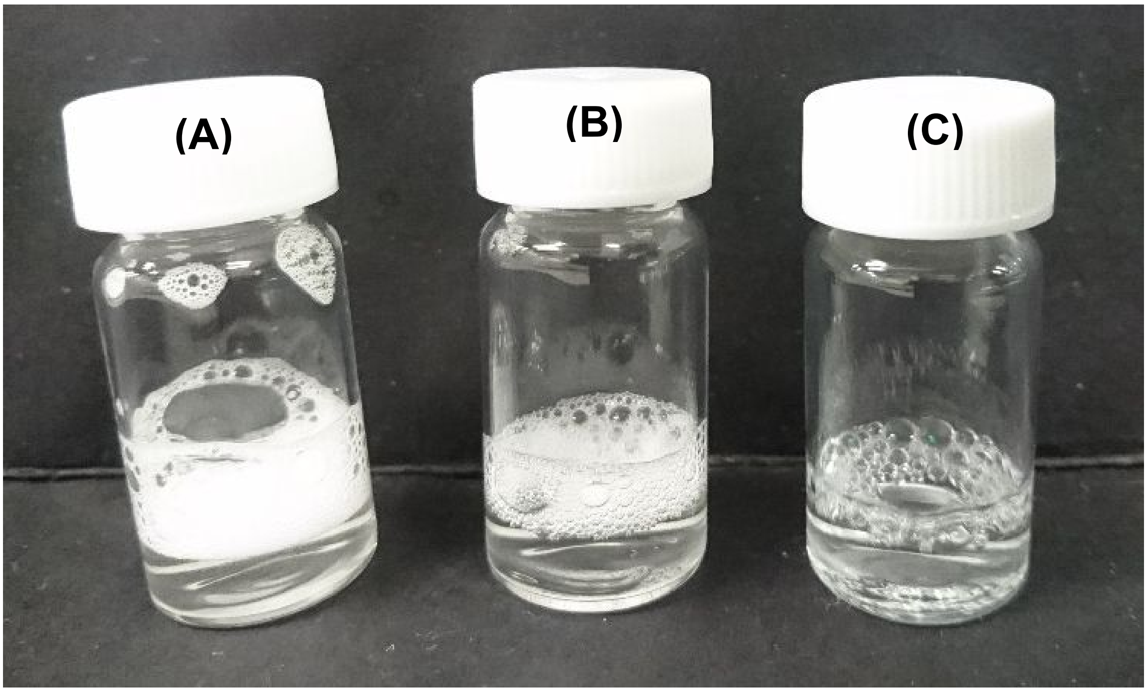

Figure S2. Digital photographs of foams produced after subjecting 0.50% w/w copolymer dissolved in an ethanol-water solution containing 53% w/w ethanol to agitation using a vortex mixer: (A) P(TMHA$_{0.5}$-*stat*-PEGA), (B) P(TMHMA$_{0.5}$-*stat*-PEGMA), and (C) PTMHA$_{0.6}$-*block*-PPEGA.

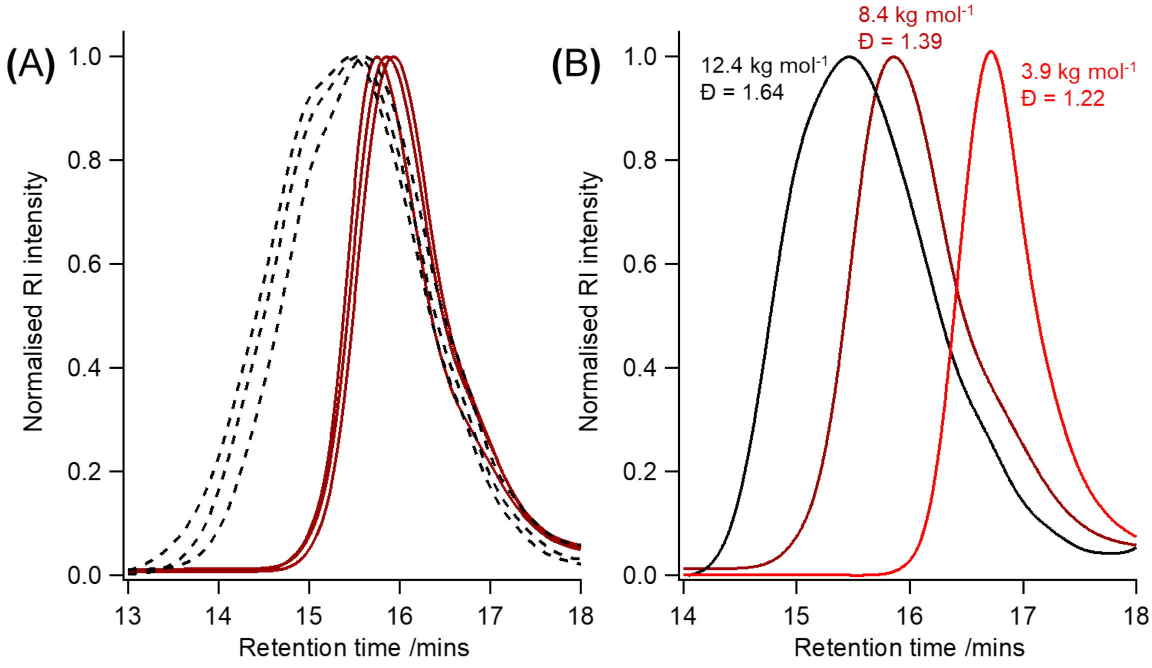

Figure S3. GPC curves recorded for: (A) three P(TMHA$_{0.4\text{-}0.6}$-*stat*-PEGA) copolymers (black dashed lines) and three R-P(TMHA$_{0.4\text{-}0.6}$-*stat*-PEGA) copolymers (red); (B) R-P(TMHA$_{0.5}$-*stat*-PEGA) synthesized with three different molecular weights. See Table S1 for a summary of these GPC data.

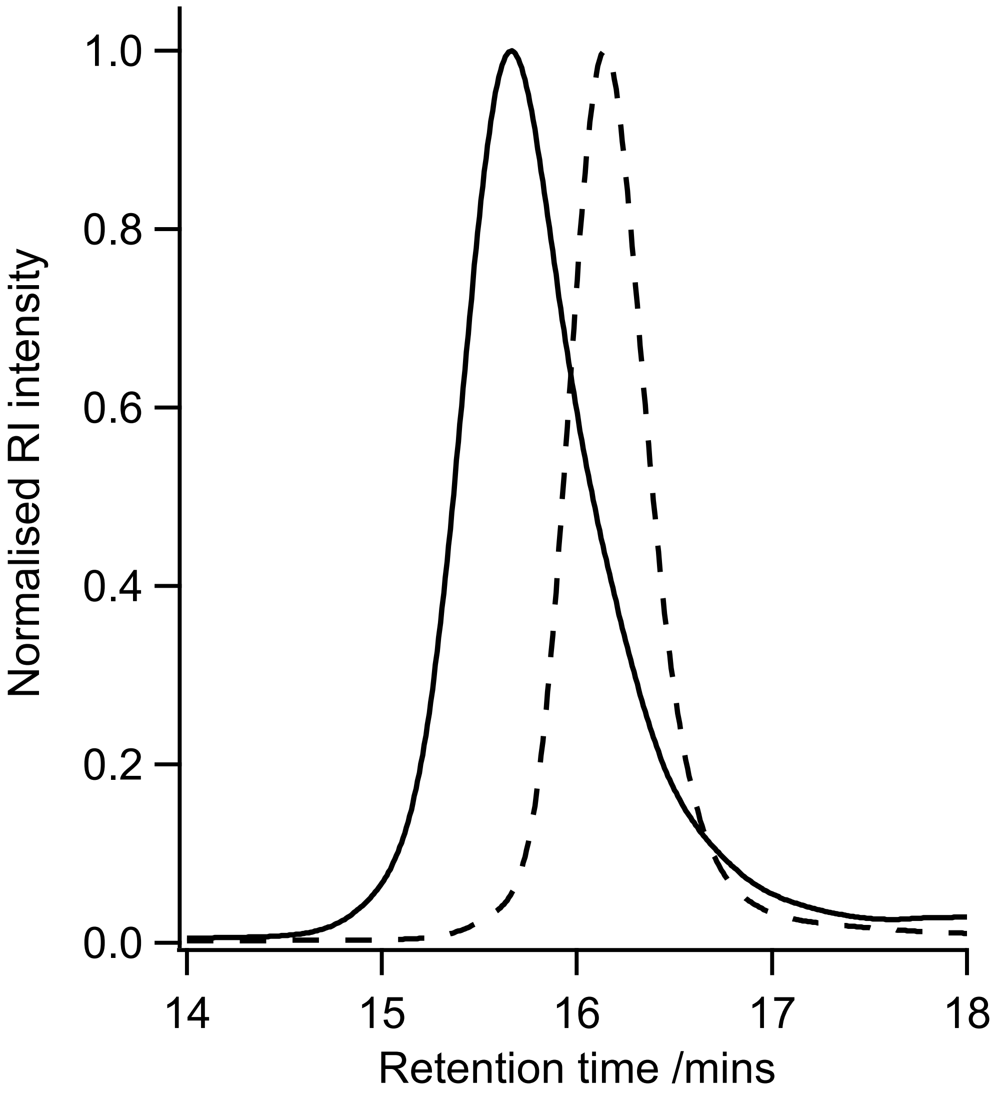

Figure S4. GPC curves obtained for the PTMHA precursor (dashed line) and the final PTMHA$_{0.5}$-*block*-PPEGA copolymer (solid line), indicating efficient chain extension.

Table S4. Parameters used to fit the SAXS data to a spherical micelle model and the mean aggregation numbers (N_{agg}) calculated using this model.

Copolymer	PEGA volume / Å³	Core radius /nm	Standard deviation /nm	Shell R_g /nm	N_{agg}
P(TMHA-*stat*-PEGA)	730	1.5	0.7	0.5	1.18
PTMHA-*block*-PPEGA	12431	6.3	1.1	2.2	91.5

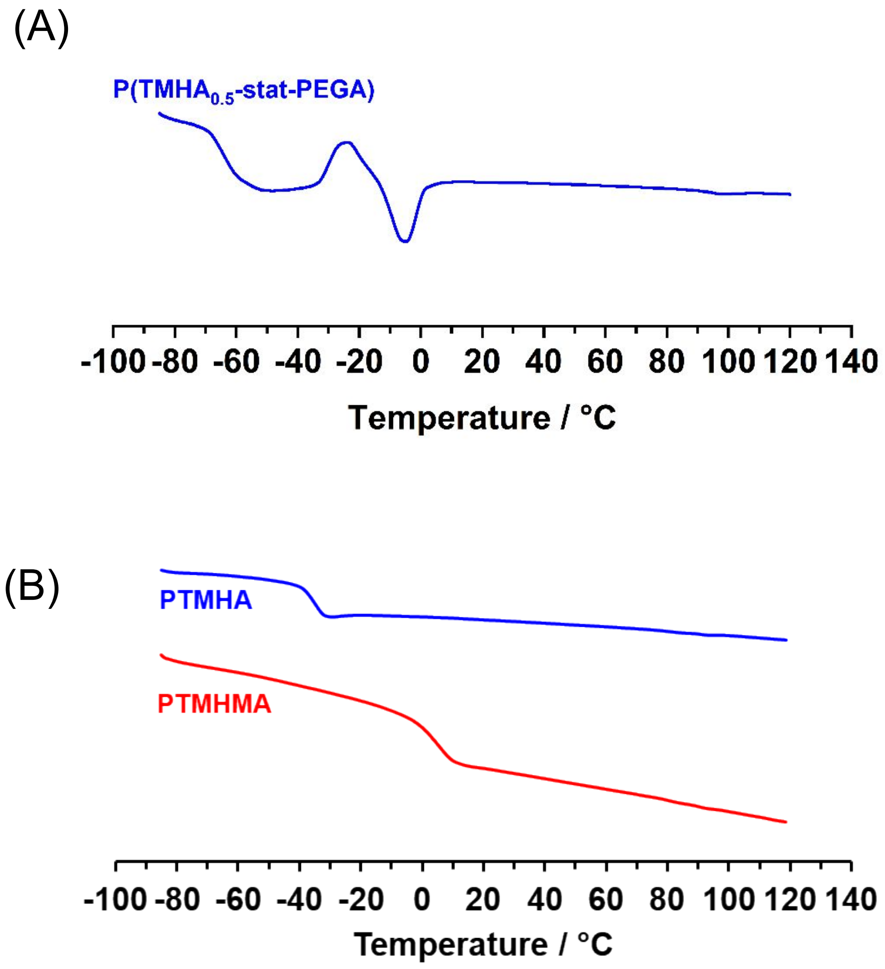

Figure S5. DSC curves recorded for (A) P($TMHA_{0.5}$-*stat*-PEGA) and (B) poly(trimethylhexyl acrylate) (PTMHA) and poly(trimethylhexyl methacrylate) (PTMHMA) homopolymers prepared by FRP.

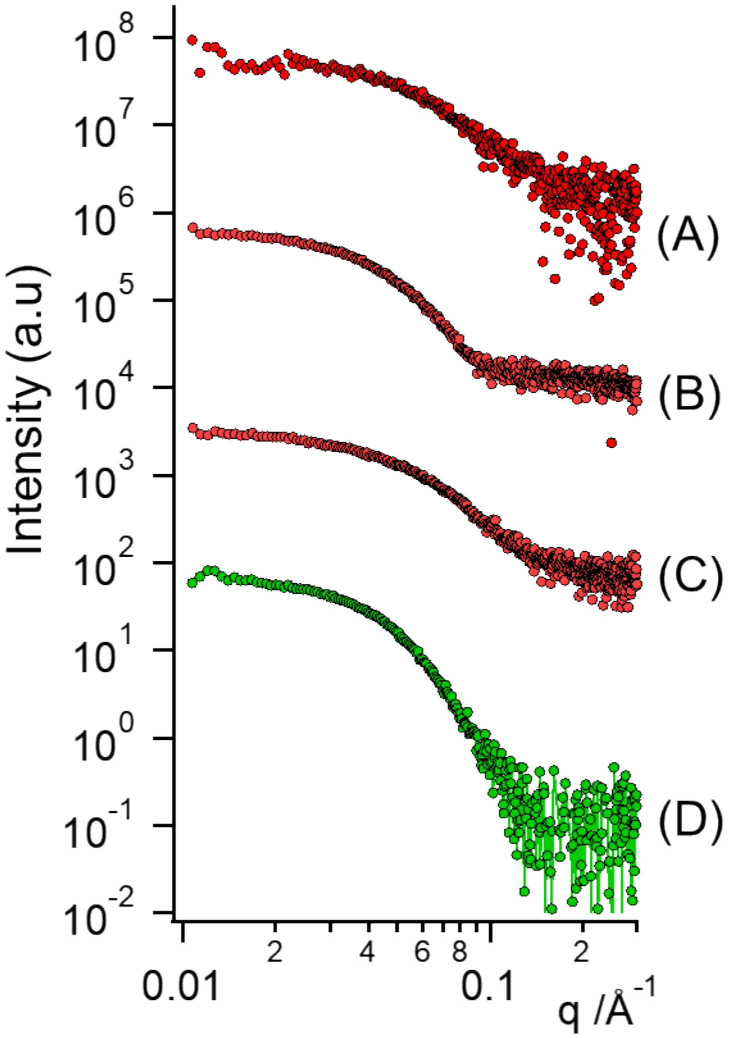

Figure S6. SAXS patterns recorded for the following statistical copolymers: (A) R-P(TMHA$_{0.5}$-*stat*-PEGA), (B) P(LA$_{0.5}$-*stat*-PEGA), (C) P(^{i}OA$_{0.5}$-*stat*-PEGA), and (D) P(TMHMA$_{0.5}$-*stat*-PEGMA). The *q* minima are comparable to that for P(TMHA$_{0.5}$-*stat*-PEGA), which indicates similar micellar radii and hence suggests unimolecular micellization.

Video S1 is attached as a separate file (Sanitiser Foaming.mp4)
